# Drivers of Continued Fitness Short Video Usage: An Integrated Model of Technology Acceptance and Health Beliefs

**DOI:** 10.3390/bs16081335

**Published:** 2026-08-03

**Authors:** Lingli Tang, Shuming Zou, Xiaoting Chen, Qing Xie, Huimin Wang, Liuliu Fang, Shiyi Yu

**Affiliations:** 1 School of Digital/Institute of Digital Publishing, Wuhan University of Technology, Wuhan 430070, China; lilytang@whut.edu.cn (L.T.); 351549@whut.edu.cn (S.Z.); felixxq@whut.edu.cn (Q.X.); 360225@whut.edu.cn (H.W.); 360211@whut.edu.cn (L.F.); 2 School of Chinese Language and Literature, Hubei University, Wuhan 430062, China; 3 School of Law, Humanities and Sociology, Wuhan University of Technology, Wuhan 430070, China; useaysy@163.com

**Keywords:** fitness short videos, continued usage, UTAUT2, Health Belief Model, Theory of Planned Behavior, habit, loss aversion

## Abstract

Introduction: Fitness short videos have become an increasingly popular form of digital health communication, yet the factors influencing users’ continued usage remain insufficiently understood. This study integrates the Unified Theory of Acceptance and Use of Technology 2 (UTAUT2), the Health Belief Model (HBM), and the Theory of Planned Behavior (TPB) to examine the determinants of users’ continuous intention and actual use of fitness short videos. Methods: A cross-sectional questionnaire survey was conducted using a convenience sampling approach among Chinese fitness short video users. A total of 550 valid questionnaires were collected. Structural equation modeling (SEM) and bootstrapping analysis were employed to examine the proposed research model and test the hypothesized relationships among constructs. Results: The results indicate that performance expectancy, hedonic motivation, habit, perceived susceptibility, perceived severity, perceived benefits, perceived barriers, perceived behavioral control, and behavioral intention significantly influenced users’ continued fitness short video usage. Habit exerted significant effects on both behavioral intention and actual use behavior. In contrast, attitude, subjective norm, and self-efficacy did not show significant direct effects. Conclusions: This study extends current understanding of continuous fitness short video usage by integrating technology acceptance, health belief, and planned behavior perspectives into a unified framework. The findings provide theoretical insights into digital health behavior and offer practical implications for the design of fitness short-video platforms and digital health intervention strategies.

## 1. Introduction

With an increase in the number of people who are sedentary and a decline in their health, people’s health awareness has risen and they are more eager to participate in exercise and fitness activities (Piercy et al., 2018; White et al., 2017). Globally, the rate of physical inactivity is still relatively high; therefore, people’s concern about health has increased and promoting exercise has received much more attention (Guthold et al., 2018). As one of the essential elements in today’s life, physical activity has been well known to improve health. Engagement in physical activity has promoted the development of all aspects of one’s health and improved flexibility, balance and strength (Park et al., 2022; Piercy et al., 2018; Vella et al., 2023). Recent studies have also shown that people who exercise regularly are healthy and have had some good effects on heart health; therefore, they are recommended for the prevention of chronic diseases (Piercy et al., 2018). Taken together, these findings indicate that although public awareness of health has increased, there remains a growing need for accessible and convenient approaches that can effectively promote regular physical activity.

With the transition to the post-pandemic new normal, public awareness of health has increased, prompting many fitness institutions to offer integrated online and offline exercise programs. Recent studies suggest that the web-based fitness environment has evolved beyond a temporary response to the pandemic and has become an independent and sustainable digital fitness ecosystem. Xuan et al. (2024) have found that the usability and perceived value of digital fitness platforms are related to how long users stay on the platform and pay for it. Xiao et al. (2026) have found that the affordance of visibility in short fitness videos, such as algorithm suggestions and social activities, can motivate people to exercise. Garcia (2024) also noted that, recently, fitness groups on social media have begun to offer people easy and inexpensive ways to stay active. Technology affordances can help to build a good user experience and make people want to continue using the short-video application for health information (Zhang & Xu, 2020).

Fitness-related content has become an increasingly important category on short-video and social media platforms. Previous studies have shown that watching fitness videos on platforms such as TikTok can promote health awareness and support participation in physical activity (Garcia, 2024). Furthermore, users’ perceived value of fitness-related video content and platform usability can significantly influence their continued usage intentions (Xuan et al., 2024; M. Yu et al., 2025). Research has also demonstrated that technological affordances, such as visibility, recommendation algorithms, and social interaction features, can enhance user engagement and motivate exercise participation (Xiao et al., 2026). These findings suggest that fitness short videos have become an important digital health communication tool and highlight the need to understand the factors that drive their continued use. However, attracting users to fitness short videos does not necessarily translate into sustained engagement or long-term exercise participation. Evidence from digital health and home-based exercise research suggests that maintaining exercise adherence remains a persistent challenge, with many users discontinuing exercise programs after initial adoption despite the availability of online support and intervention tools (Lang et al., 2022). This challenge has been reported across different countries and digital health contexts, suggesting that sustaining engagement in technology-supported exercise programs remains a global concern rather than a context-specific issue (Lang et al., 2022). Although fitness short videos provide an accessible and low-cost channel for health communication, the effectiveness of such interventions ultimately depends on whether users remain engaged over time and translate media exposure into sustained exercise behavior. Understanding why users continue to engage with fitness short videos is therefore important, because the potential health benefits of such content can only be realized when users sustain their participation and translate media exposure into regular exercise behavior.

Although there have been many studies on the initial adoption of short-video platforms, relatively little research has examined the psychological mechanisms underlying continued fitness short video usage. Most previous studies have focused on general social media applications or the initial acceptance of digital technologies (Limayem et al., 2007; Venkatesh et al., 2012), whereas the factors that sustain long-term engagement in fitness-related short-video environments remain insufficiently understood. Given the high dropout rates associated with home-based exercise, understanding whether users’ technology perceptions, health beliefs, and behavioral intentions can promote continued engagement has become increasingly important. Developing a more comprehensive explanatory framework may help researchers and practitioners better understand the mechanisms underlying sustained engagement with fitness short videos and design more effective digital health interventions. To address this gap, the present study integrates UTAUT2, the Health Belief Model (HBM), and the Theory of Planned Behavior (TPB) to provide a more comprehensive explanation of continued fitness short video usage behavior. Therefore, this study aims to explore the factors that drive users’ transition from sporadic viewing to sustained fitness participation and regular exercise behavior (Cheung & Lee, 2010; Taylor & Todd, 1995).

Most existing research on the acceptance of digital health technology is based on the theoretical foundation of the technology acceptance model (TAM) or the Unified Theory of Acceptance and Use of Technology (UTAUT) (Limayem et al., 2007; Rosenstock, 1974; Venkatesh et al., 2012). The above models have mainly concentrated on the spread of functional technology and have not considered people’s sense of health or social-psychological barriers (Angosto et al., 2020; Li et al., 2025). UTAUT2 has hedonic motivation and does not contain the section on health-related risk perception. To address the above theoretical deficiencies, this study will be based on the three main theoretical systems: UTAUT2, Health Belief Model (HBM) and Theory of Planned Behavior (TPB). Compared with the original UTAUT2 model, this paper adds health-related risk assessments (perceived susceptibility, perceived severity, perceived benefits, perceived barriers, self-efficacy) from the HBM and social-psychological constraints (attitude, subjective norm, perceived behavioral control) from the TPB to better represent the specific situation of digital fitness.

To validate the proposed research model, data were collected from 550 regular users of fitness short videos. Structural equation modeling (SEM) and bootstrapping analysis were employed to examine the hypothesized relationships and mediation effects among the study variables. This study makes three main contributions. First, it integrates the technology acceptance theory, the Health Belief Model, and the Theory of Planned Behavior into a unified framework for explaining continued fitness short video usage. Second, the findings suggest that habit influences both behavioral intention and actual use, indicating a dual-path mechanism of habitual behavior. Third, the results show that self-efficacy, attitude, and subjective norm did not significantly predict behavioral intention in the fitness short-video context, providing new evidence regarding the applicability of classical behavioral theories in digital fitness environments.

The rest of this paper is structured as follows: Section 2 will present a summary of the existing research on digital health communication and fitness short video usage behavior. Section 3 is the theoretical basis (UTAUT2, HBM and TPB), the research hypotheses, and the construction of the integrated theoretical model. Section 4 is the research method, which will present the measurement scale and data collection plan. Section 5 is the empirical analysis part and the model test results are presented here. The main results of Section 6 are presented below. Section 7 is the theoretical and practical significance of the study. Finally, Section 8 is the conclusion and discussion section of this paper, along with suggestions for further study.

## 2. Literature Review

### 2.1. Digital Health Communication and Fitness Short Videos

In line with the evolution of digital health communication (DHC), public health promotion has shifted from traditional one-way broadcasts to interactive, technology-driven platforms. Fitness short videos have emerged as a prominent medium within this shift, offering convenient, low-cost at-home exercise instruction tailored to modern busy schedules (Park et al., 2022; Vella et al., 2023; X. Yu et al., 2024). By utilizing rich audiovisual elements—such as background music, verbal narration, and movement demonstrations—these brief interventions effectively lower the barrier to exercise participation and enhance users’ health cognition. Consequently, digital health platforms are increasingly viewed as transformative communication systems that leverage technology to promote healthy living.

Beyond the basic format, user engagement with fitness videos is heavily driven by platform affordances and the social influence of content creators (Garcia, 2024; Xiao et al., 2026; M. Yu et al., 2025). The first studies on online exercise interventions reported that digital fitness platforms can effectively support exercise participation and enhance users’ willingness to engage in physical activity. For example, Zhang and Xu (2020) found that perceived usefulness and social support significantly influenced users’ continued use of fitness applications, while Yang and Koenigstorfer (2021) demonstrated that motivation-related and gamification-related app features positively affected fitness app usage intentions (Przybylski et al., 2010; Yang & Koenigstorfer, 2021). Furthermore, fitness influencers play a crucial role in shaping exercise attitudes on platforms like YouTube and TikTok. The specific traits of these influencers—such as physical attractiveness and trustworthiness—serve as powerful motivational cues that encourage viewers to participate in at-home workouts (Durau et al., 2022; H. M. Kim et al., 2023; Sun et al., 2024; Zhang et al., 2017).

Despite the extensive literature on the initial adoption of digital health tools and the motivational impact of influencers, the psychological mechanisms underlying long-term, continued usage remain underexplored. The persistently high dropout rate associated with digital health interventions indicates that initial motivation and platform content alone are insufficient to guarantee sustained behavioral change. To understand the transition from occasional viewers to regular practitioners, a single theoretical lens is inadequate. Therefore, integrating technology acceptance theories with health-belief and social psychology frameworks is essential to comprehensively address the dynamics of continuous fitness short-video consumption.

### 2.2. Fitness Short Video Usage Motivation and Behavior

Different motives for using digital media significantly influence user behavior. Research based on the uses and gratifications theory demonstrates that user motivations are often more prominent drivers of short-video consumption (e.g., TikTok) than stable personality traits (Cuesta-Valiño et al., 2022; Omar & Dequan, 2020). While specific motives vary across demographics—such as middle-aged and older people’s use of short videos to obtain information (X. Yu et al., 2024)—and across different platforms (Lin et al., 2017), these intrinsic utilitarian, hedonic, and social needs form the fundamental basis for initial platform adoption.

Motivation of users is an internal force that directly drives their external behavior (Flecha-Ortíz et al., 2021). In the context of fitness short videos, previous studies have suggested that users are primarily motivated by information acquisition, entertainment, social interaction, and self-improvement (Omar & Dequan, 2020; X. Yu et al., 2024). Consistent with this view, Rosli and Dahlan (2021) found that users continue to use short-video applications for health information specifically because of these needs, alongside convenience, accessibility, and the perceived usefulness of the content. However, translating these internal motivations into actual, sustained exercise behaviors is complex. These motivations influence users’ willingness to engage with fitness-related content, but their continued engagement is also heavily influenced by contextual factors, such as platform characteristics, information quality, social environments, and individual user characteristics (X. Yu et al., 2024; M. Yu et al., 2025).

Based on the above studies of user motivation, research on the behavior of fitness short videos can be divided into three main directions: content features (e.g., production forms and entertainment value) (Omar & Dequan, 2020; Zhang et al., 2017), specific user profiles and their corresponding motives (Omar & Dequan, 2020; X. Yu et al., 2024), and general adoption behaviors. Although research has shown that utilitarian, hedonic, and social needs drive initial use, the existing literature lacks a systematic framework to explain how these initial motivations lead to continuous usage behavior. To address this gap, the purpose of this paper is to solve these problems through a multi-theoretical integration that connects technology adoption theories with health beliefs.

### 2.3. Application of Multi-Theoretical Integration in Digital Content Continuous Usage Research

Given that the specific theoretical constructs of the Unified Theory of Acceptance and Use of Technology 2 (UTAUT2), the Health Belief Model (HBM), and the Theory of Planned Behavior (TPB) will be detailed in the hypothesis development section, this part will mainly focus on the application of multi-theoretical integration in the field of continuous digital content use. Recently, scholars have started to study the long-term use of digital health products, such as short videos, mHealth applications and wearable devices, and have determined that they involve various decisions. One theory is insufficient to address all the issues in user behavior. Therefore, in recent years, integrating the theory of technology acceptance with theories of health behavior has been promoted to address the need to understand why some people continue to use health apps and other technologies.

Most studies have found some problems in the integration of technology, and these problems may be associated with changes in the digital economy. UTAUT (or UTAUT2) in conjunction with HBM is a typical way to study digital health platforms. Sun et al. (2024) employed UTAUT2 and HBM to study the continuous use intention of women’s health WeChat public accounts, and it was found that adding a health threat assessment improved the prediction accuracy of the model for continuous use. Guo and Chen (2025) used UTAUT and HBM to study the acceptance of wearable devices by chronic disease patients, and it was found that both the expectation of technological performance and the perception of health risk promoted use. In recent years, the combination of HBM and TPB has been applied to physical activity studies. Qiao et al. (2021) have successfully employed the combination of HBM and TPB to examine beliefs about physical activity; it was found that when integrating health risk appraisal with behavioral control and subjective norms, the intention–behavior gap in exercise behavior can be addressed.

Based on the above references, it is both reasonable and very necessary to integrate UTAUT2, HBM and TPB to explore the continuous usage behavior of fitness short videos. Fitness short videos have two characteristics: they are entertainment for the public and health promotion at the same time. UTAUT2 can address the issue of technology acceptance and show that, for example, people are motivated to use apps because they are entertaining or easy to operate. Media consumption is not the same as fitness behavior. HBM considers the need for good health, explains why people are interested in learning about fitness (such as fear of being overweight), and so on. Finally, TPB adds subjective norms and perceived behavioral control to address the problem of only “watching” and not actively “doing”; therefore, it can be used to motivate and maintain exercise.

Therefore, through the integration of the three theories, we can correct the deficiencies of single-theory views that are either too focused on the technology medium or too concentrated on the physical health result. The three theories combined provide a comprehensive view of the entire behavioral chain, that is to say, how people begin to use the platform, form beliefs about health, and ultimately act in accordance with these beliefs; thus, a solid theoretical foundation for the research design and hypothesis testing of this paper has been established.

## 3. Model Development and Hypotheses

### 3.1. Unified Theory of Acceptance and Use of Technology 2 (UTAUT2)

Venkatesh et al. (2012) proposed the Unified Theory of Acceptance and Use of Technology 2 (UTAUT2), which is a broad system that explains why people adopt and continue to use digital technologies. UTAUT2 is an expansion of the original UTAUT model for organizations, which has added consumer-oriented factors such as hedonic motivation (HM) and habit (HB), and it is now known that performance expectancy (PE), HM, and HB are all involved in predicting behavior intention and actual use behavior (Venkatesh et al., 2012). UTAUT2 has been used extensively in many studies of mobile applications, social media platforms and digital health technology due to its strong explanatory power (Wang et al., 2026), and it is especially suitable for the situation of fitness short videos because it can grasp both the instrumental (utilitarian) and experiential values of technology use.

It is to be noted that two constructs from the original UTAUT2 model were not used in this study: “Price Value” did not fit because fitness short video content is generally free of charge, and “Effort Expectancy” was also excluded due to the very-low technical threshold of mobile video platforms (e.g., simple vertical scrolling).

Performance expectancy is the degree to which people believe that the fitness short video will help them improve their physical condition, gain knowledge about fitness, etc. Most studies have long since shown that performance expectancy is a strong driver for people’s intentions to use new technologies (Venkatesh et al., 2012). If the users believe that the fitness short videos can help them meet their own fitness goals, they will be motivated to watch them more frequently.

Hedonic motivation is the sense of pleasure and happiness people get from watching fitness short videos. Given that short-video platforms are for entertainment, they will attract people to start watching and thus continue to watch them over time (Orji et al., 2012; Venkatesh et al., 2012).

UTAUT2 also indicates that habit can promote both the intention of behavior and actual use behavior. Habit is how frequently people perform behaviors automatically after learning from or experiencing things many times (Cheung & Lee, 2010; Venkatesh et al., 2012). Habit is an automatic behavior that forms over time through repeated use of a technology; it is not one of the two cognitive factors, performance expectancy and hedonic motivation. After watching many fitness short videos, users may start to have habitual patterns in how they watch, share, or follow exercise content. These regular habits can give people a sense of purpose and thus inspire them to continue using the fitness short-video service. At the same time, habit can directly motivate people to behave in a certain way without having to think about it consciously, so they will automatically do so even if their intention is relatively weak. Therefore, both the deliberate cognitive process (namely, usage intention) and the unintentional automatic response (namely, habit) work together to drive the continuous use of fitness short videos.

Accordingly, this study proposes the following hypotheses based on UTAUT2:

**H1a.**  *Performance expectancy positively influences users’ intention (BI).*

**H1b.**  *Hedonic motivation positively influences users’ intention (BI).*

**H1c.**  *Habit positively influences users’ intention (BI).*

**H1d.**  *Users’ intention (BI) positively influences their actual usage behavior (UB).*

**H1e.**  *Habit positively influences users’ usage behavior (UB).*

### 3.2. Health Belief Model (HBM)

The Health Belief Model (HBM) was proposed by Rosenstock (1974), and is a well-known theory that aims to explain the reason why people engage in health behavior, which believes that people will only engage in such behavior if they perceive the risk of illness and think that it is worthwhile to take the time and effort to do so (Rosenstock, 1974). The five main factors that determine people’s intention to engage in health-related behavior according to the model are perceived susceptibility (PS), perceived severity (PSev), perceived benefits (PB), perceived barriers (PBar) and self-efficacy (SE) (Angosto et al., 2020; J. Kim & Park, 2012). Meta-analysis has found that there is an asymmetry in the model, and often the inhibitory effect of negative barriers (PBar) is greater than the promotional effect of positive benefits (PB) (Janz & Becker, 1984). Self-efficacy has long been one of the main indicators of people’s health behavior, but whether it can work in the “foolproof” and “low-threshold” environment of short videos is still unknown; in such circumstances, the ease of imitation might reduce the effect of high self-confidence.

HBM is more about the health perspective than the technology-oriented acceptance model, and thus can provide a better reason for the use of fitness-related digital content by people. Considering the background of fitness short videos, people may be motivated by worries about being physically inactive, the development of lifestyle-related diseases, or the wish for a healthier life in old age. When people feel that they are at risk of poor health and believe that this will lead to serious consequences, they will be more willing to use easy-to-access health-promoting resources (Janz & Becker, 1984). Fitness short videos can show how to do it visually and are convenient and have a low barrier to entry; thus, people are more willing to participate in healthy behavior.

Perceived benefits are the beliefs of people that using fitness short videos will lead to some good health effects, such as gaining knowledge about exercise, being motivated to exercise, and exercising more efficiently (Bandura, 1997). At the same time, there are also perceived obstacles, such as a lack of time, no personalized services, and doubt about the accuracy of the information (Conner & Armitage, 1998). Self-efficacy is the sense of an individual’s ability to carry out exercise according to instructions and to continue exercising regularly; it is also a reason for exercise (Bandura, 1997).

Based on the Health Belief Model, this study proposes the following hypotheses:

**H2a.**  *Perceived susceptibility positively influences users’ intention (BI).*

**H2b.**  *Perceived severity positively influences users’ intention (BI).*

**H2c.**  *Perceived benefits positively influence users’ intention (BI).*

**H2d.**  *Perceived barriers negatively influence users’ intention (BI).*

**H2e.**  *Self-efficacy positively influences users’ intention (BI).*

### 3.3. Theory of Planned Behavior (TPB)

As proposed by Ajzen (1991), the Theory of Planned Behavior (TPB) holds that the main reasons people will behave a certain way can be broken down into three parts: attitude towards the behavior, subjective norm (or perceived social pressure), and perceived behavioral control (how easy or difficult it is to perform the behavior). Attitude is a person’s judgment of the behavior, subjective norm is the perceived social pressure to participate in or not to participate in this behavior, and perceived behavioral control is the perceived ease with which the behavior can be carried out. Widely used in research on health behavior, media use and technology adoption, the Theory of Planned Behavior (TPB) has shown good explanatory power in many behavioral contexts (Conner & Armitage, 1998) and can be applied to investigate the formation of intention in socially embedded media environments such as short-video platforms through the combination of social influence and perceived control.

Attitude refers to users’ overall evaluation of using fitness short videos for fitness and health purposes. Individuals who perceive fitness short videos as useful, enjoyable, and beneficial are more likely to develop positive attitudes toward their use, thereby increasing their behavioral intention to engage with such content (Ajzen, 2002).

Subjective norm refers to the perceived social pressure from important others, such as family members, friends, and peers. Given the social nature of short-video platforms, social endorsement and normative expectations may encourage users to view and engage with fitness-related content (Taylor & Todd, 1995).

Perceived behavioral control reflects individuals’ perceptions of their ability to use fitness short videos, including the availability of time, resources, and necessary skills. When users perceive that they have sufficient resources, opportunities, and abilities to use fitness short videos, they are more likely to develop stronger behavioral intentions (Taylor & Todd, 1995).

Based on the Theory of Planned Behavior, the following hypotheses are proposed:

**H3a.**  *Attitude positively influences users’ intention (BI).*

**H3b.**  *Subjective norm positively influences users’ intention (BI).*

**H3c.**  *Perceived behavioral control positively influences users’ intention (BI).*

**Figure 1:**
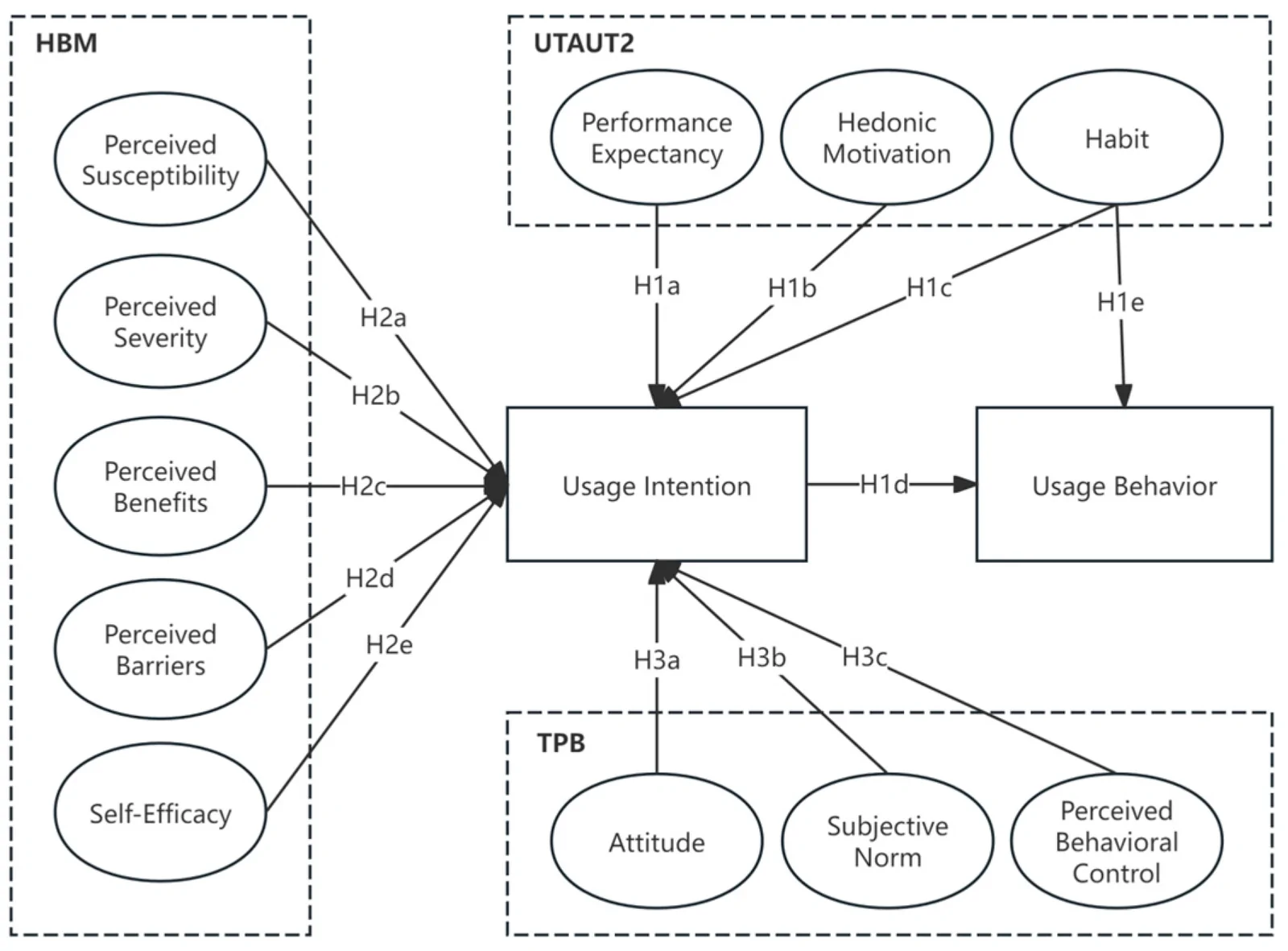
The integrated theoretical model for fitness short video continued usage. HBM: Health Belief Model; UTAUT2: Unified Theory of Acceptance and Use of Technology 2; TPB: Theory of Planned Behavior; H1a–H3c represent the proposed research hypotheses.

As shown in Figure 1, based on the hypotheses proposed above, the theoretical framework was established.

## 4. Materials and Methods

### 4.1. Questionnaire Design and Measurement Scales

To guarantee the reliability and validity of the measuring tool in this paper, all items are drawn from well-known scales in previous studies and then adjusted according to the actual conditions of fitness short-video consumption. To ensure linguistic equivalence, considering the fact that the original scales are in English and the target respondents are Chinese, translation and back-translation procedures were strictly followed. Furthermore, prior to formal data collection, the survey instrument was pilot-tested on a small sample of non-participants (*N* = 30 regular fitness short video users) to evaluate its comprehension, logical flow, and cultural appropriateness. Based on the feedback gathered during this pre-test, minor modifications were made to the phrasing of certain items to eliminate ambiguities and ensure that the wording was natural and easily understood by the Chinese target audience. The two parts of the questionnaire are as follows: The first part introduced the characteristics of the surveyed group and basic usage situations of fitness short videos, such as gender, age, education level, how often they watched them, etc.

The second part is to measure the 13 main latent variables of the integrated theoretical framework using a 5-point Likert scale, and the options range from 1 (“strongly disagree”) to 5 (“strongly agree”). Specifically, the measurement items for performance expectancy (PE), hedonic motivation (HM) and habit (HB) were taken from the UTAUT2 model put forward by Venkatesh et al. (2012) and Limayem et al. (2007). The constructs of the Health Belief Model (HBM), that is to say, perceived susceptibility (PS), perceived severity (PSev), perceived benefits (PB) and perceived barriers (PBar), were measured based on modified scales from Rosenstock (1974) and Champion (1984). Self-efficacy (SE) is based on the original work by Bandura (1997) and Champion (1999). Additionally, the variables based on the Theory of Planned Behavior (TPB), namely attitude (ATT), subjective norm (SN) and perceived behavioral control (PBC), were adapted from Ajzen (1991) and Taylor and Todd (1995). Finally, the outcome variables of behavioral intention (BI) and actual use behavior (UB) were taken from the standard items provided by Venkatesh et al. (2003, 2012) and Ajzen (1991). The above constructs and their corresponding items are presented in Table 1.

### 4.2. Data Collection and Quality Control

Wenjuanxing (Questionnaire Star) is a good tool and widely used online survey platform in China that was employed for this purpose. Given the exploratory nature of this study, a non-probability convenience and snowball sampling approach was utilized to recruit a diverse sample of fitness short video consumers. The survey link was distributed across various online fitness communities, student groups, and social networking platforms (e.g., WeChat and QQ).

To ensure transparent reporting and validity, explicit eligibility criteria were established. The inclusion criteria required participants to be regular Internet users, have experience watching fitness short videos, and provide voluntary consent. Conversely, the exclusion criteria eliminated individuals lacking recent viewing history (within six months), those failing attention checks, and those with unrealistically short completion times.

Prior to data collection, an a priori sample size estimation was conducted to determine the minimum number of participants needed to detect a statistically significant effect with a 95% confidence level (α = 0.05). Based on the widely accepted guideline for structural equation modeling (SEM) requiring at least 10 observations per measurement item, our model consisting of 44 items required a minimum baseline of 440 respondents. Furthermore, an a priori power analysis calculated for a medium effect size with a targeted 95% statistical power (for a structural model with 11 primary predictors influencing behavioral intention) yielded a minimum threshold of approximately 200 participants. Thus, our targeted sample was explicitly set above 440 to guarantee robust parameter estimation.

The strategy to determine that selected participants met the established criteria relied on automated platform functions and post-collection screening. To ensure the quality and accuracy of the empirical data, some stringent quality checks have been carried out on the collected information. First, a screening question (“Have you watched fitness short videos in the past six months?”) was placed at the start of the questionnaire; those who replied “No” were immediately taken to the end of the survey. Second, attention-check questions were added to the main construct section to find and discard random answers. Thirdly, the system will record the time a respondent finishes filling out the questionnaire, and any that are completed too quickly (e.g., in less than 60 s) will be regarded as invalid and will not be included in the final data. Furthermore, regarding missing values, all questions in the online survey were configured as mandatory, thereby effectively preventing item non-response. Any partially completed questionnaires (e.g., dropouts) were entirely excluded from the dataset using listwise deletion. Additionally, the dataset was screened for statistical outliers to ensure robust parameter estimation. Because the observed variables were measured on a bounded 5-point Likert scale, traditional univariate outliers were theoretically constrained and not applicable. Instead, multivariate outliers across the 13 latent variables were assessed using the Mahalanobis distance (D^2^) criterion with a conservative threshold of *p* < 0.001. Through this assessment, 15 cases (representing approximately 2.6% of the initially completed surveys) were flagged. Upon inspection, the plausible reason for their exceptionality was highly contradictory response behaviors across conceptually related items, likely stemming from respondent fatigue. Consequently, these cases were excluded.

Finally, after removing invalid answers that were the same as the previous one or failed the attention check, or were identified as multivariate outliers, a total of 550 valid questionnaires were obtained for the rest of the analysis, and this constituted the final sample (*N* = 550) used in Section 5. This final sample size (*N* = 550) comfortably exceeds both mathematically estimated minimum requirements, ensuring sufficient statistical power for the study.

### 4.3. Ethical Statement

Ethical review and approval were waived for this study in accordance with the local legislation and institutional requirements (Article 32 of the Measures for Ethical Review of Life Sciences and Medical Research involving Human Beings of China; detailed information can be found at https://www.gov.cn/zhengce/zhengceku/2023-02/28/content_5743658.htm, accessed on 15 August 2025), as it did not entail clinical trials or manipulations involving humans or animals.

### 4.4. Analytical Strategy

In this study, structural equation modeling (SEM) was selected as the primary analytical strategy, implemented using SPSS 26.0 and AMOS 26.0. The choice of SEM is explicitly justified by several methodological advantages that align with the complexity of our research objectives. First, SEM is robust in simultaneously estimating a series of interrelated dependence relationships, which is essential for testing the complex, multi-path integrated framework encompassing UTAUT2, HBM, and TPB constructs (Anderson & Gerbing, 1988; Hair et al., 2010). Second, unlike traditional multiple regression, SEM explicitly accounts for measurement error within the observed variables, thereby providing a more rigorous and accurate evaluation of construct validity and theoretical reliability (Hair et al., 2010). Finally, SEM facilitates the robust examination of both direct effects and indirect mediating mechanisms. By integrating SEM with bootstrapping techniques, this study can accurately isolate the mediating role of behavioral intention and the dual-path influence of habit, enabling a comprehensive deconstruction of users’ continuous usage behavior (Hayes, 2013).

## 5. Results

SPSS 26.0 and AMOS 26.0 were used for empirical analysis and model testing. In line with the two-step method proposed by Anderson and Gerbing (1988), the evaluation process will first assess the measurement model and then evaluate the structural model.

### 5.1. Demographic Profile and Descriptive Statistics

In this study, SPSS 26.0 was utilized to conduct comprehensive descriptive statistical analysis on the demographic characteristics of the sample. This included the calculation of frequencies, percentages, means, and standard deviations to establish the respondent profile. Furthermore, it was used to assess the initial internal consistency and reliability of the measurement scales prior to structural modeling.

Table 2 provides a detailed breakdown of the demographic characteristics and usage patterns of the respondents (*N* = 550). Based on the proportion of women and men, 56.4% are women and 43.6% are men. The main group of people who filled out the survey (78.4%) were in their twenties and thirties; this group has a strong interest in online fitness. About 53.8 per cent of the people enjoy such content 2–4 times a week. In addition, 72.9 per cent of the users said they watch a single video for less than 30 min, so the consumption is scattered.

**Figure 2:**
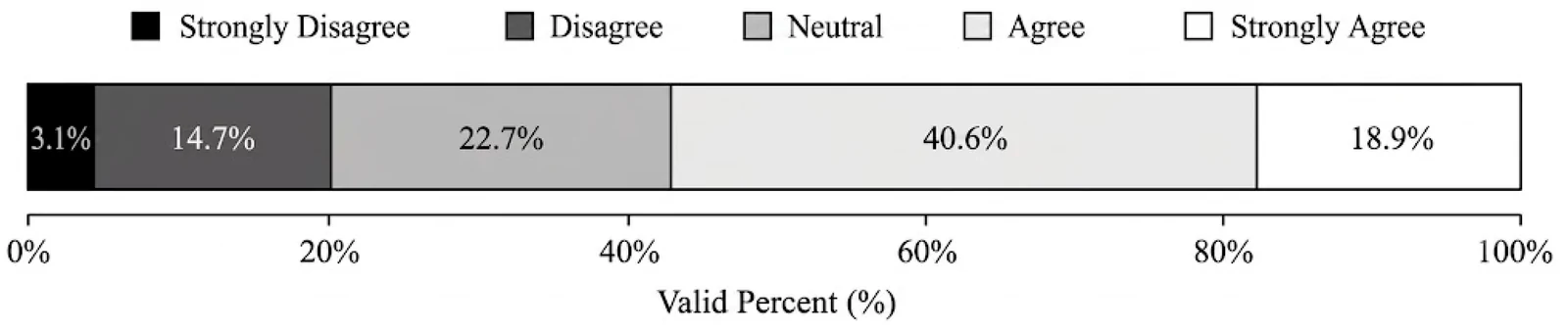
Distribution of respondents’ continuance intention towards fitness short videos (item: BI1). The stacked bar chart illustrates a predominantly positive behavioral intention among the surveyed users.

In addition to the basic demographic profile, we visualized the distribution of responses for key measurement items to better illustrate the users’ overall behavioral tendencies. As shown in Figure 2, the majority of respondents exhibited a positive intention to continue using fitness short videos.

### 5.2. Assessment of the Measurement Model

Table 3 summarizes the reliability and validity assessment results of the measurement model. First of all, the reliability and construct validity of the hypothesis will be determined by way of internal consistency and confirmatory factor analysis (CFA) in the measurement model. As shown in Table 3, the Cronbach’s α values of all 13 constructs ranged from 0.794 to 0.888, which is higher than the recommended threshold of 0.70, and they exhibit good internal consistency and scale reliability.

To examine the construct validity of the model, standardized factor loadings, composite reliability (CR) and average variance extracted (AVE) will be used. All the standardized factor loadings of the individual measurement items were between 0.672 and 0.869, exceeding the upper limit of 0.60. Appendix A is used to list the item loadings and descriptions.

As shown in Table 3, the CR values of all latent variables were greater than the 0.70 threshold (from 0.796 to 0.889), and the AVE values were all above the acceptable limit of 0.50 (from 0.523 to 0.691). In short, the above results show that the measurement model has good convergent validity.

### 5.3. Correlation Analysis

To further validate the discriminant validity of the measurement model, we scrutinized the Pearson correlation coefficients among the latent variables. This step is essential because it demonstrates that the constructs are empirically distinct rather than overlapping. If these coefficients were excessively high, it would imply that the constructs are essentially measuring the same underlying trait, which would undermine the model’s validity. In our analysis, none of the inter-construct correlations approached the critical threshold (typically suggested as 0.85), providing strong evidence that the latent variables function as unique, independent dimensions within the theoretical framework.

Table 4 presents the Pearson correlation matrix of the latent variables. As shown in the table, all of the main indicators are significant at the 0.01 or 0.05 level. Perceived behavioral control (PBC) and behavioral intention (BI) had a small positive correlation with each other (r = 0.547, *p* < 0.01). Habit (HB) is positively correlated with BI (r = 0.400, *p* < 0.01) and actual use behavior (UB) (r = 0.270, *p* < 0.01). In addition, perceived barriers (PBar) also had a small negative correlation with BI (r = −0.096, *p* < 0.05). The above correlations have been used to build a structural equation model in the next step.

### 5.4. Model Fit and Hypothesis Testing

Based on the reliability and validity tests, as well as the correlations among the model’s variables, it has been verified that the accuracy and consistency of the research data meet the requirements. In order to further explore the reasons for these changes in the variables and clarify the direct and indirect paths of the effects, structural equation modeling (SEM) was used in this study to carry out empirical analysis of the proposed model with AMOS 26.0 software.

Based on AMOS 26.0, the first construction and mapping of an integrated theoretical model of fitness short video usage intention and behavior were conducted. Then, based on the survey data, a general goodness-of-fit test of the model was conducted, and then the model was used to test the research hypotheses one by one.

#### 5.4.1. Model Construction

According to the integration of the theoretical models of UTAUT2, HBM and TPB, along with the corresponding research hypotheses, there are 13 main latent variables in this study: performance expectancy (PE), hedonic motivation (HM), habit (HB), perceived susceptibility (PS), perceived severity (PSev), perceived benefits (PB), perceived barriers (PBar), self-efficacy (SE), attitude (ATT), subjective norm (SN), perceived behavioral control (PBC), behavioral intention (BI) and actual usage behavior (UB).

Among them, PE, HM, PS, PSev, PB, PBar, SE, ATT, SN and PBC can either directly affect the intention to use fitness short videos or indirectly impact actual use behavior via the intention to use. At the same time, use intention and habit can directly affect actual behavior. AMOS 26.0 was used to test the hypotheses and validate the model in this paper.

#### 5.4.2. Model Fit Evaluation

This study selected absolute fit indices (GFI), incremental fit indices (NFI, IFI, CFI), and a parsimonious fit index (χ^2^/df) to comprehensively evaluate the model fit, and the results are summarized in Table 5.

The fit results in Table 5 indicate that the chi-square-to-degrees-of-freedom ratio (χ^2^/df) of the proposed model is 3.538, which falls within the acceptable range of <5. The CFI (0.834), TLI (0.820), and IFI (0.835) all exceed the basic fit standard of 0.80. The PNFI (0.724) and PCFI (0.770) are well above the critical value of 0.50, indicating a good parsimonious fit for the model (Hair et al., 2010; Hu & Bentler, 1999). GFI, AGFI, and NFI are all approaching the critical value of 0.80, presenting a marginal fit status.

In this study, the RMR = 0.190 did not reach the ideal critical value of 0.08, and this is to be expected for three main reasons: First, the complex model in this study combines many theories and numerous latent variables and paths to be tested, so the accumulation of residuals is very likely (Hair et al., 2010). Second, as the basis for RMR is a relatively large number of observations (*n* = 550) and the data are ordinal categorical data from a 5-point Likert scale, the traditional critical values may not be appropriate (Hu & Bentler, 1999; Zhang et al., 2024). Thirdly, the use behavior of users of fitness short videos is not the same, and thus this model may be conceptually limited to some extent. After all the indices have been assessed, it was found that the model fits well according to all these indicators. The single RMR index deviation can be reasonably explained by combining the characteristics of the model and the data, and it will not affect the overall validity of the model or the subsequent hypothesis test.

Table 6 illustrates the path verification results for the structural equation model (*N* = 550), where the standardized path coefficients (β) are reported.

### 5.5. Mediation Analysis

To further explore the mechanisms underlying the variables, this study employed the bootstrap method (with 5000 resamples) to test the mediating effects of behavioral intention between various antecedent variables and actual behavior. The evaluation criterion is as follows: if the 95% confidence interval does not contain 0, it indicates that the mediating effect is significant (Hayes, 2013).

As shown in Table 7, the indirect effects of perceived susceptibility, perceived severity, perceived benefits, perceived barriers, performance expectancy, hedonic motivation, and perceived behavioral control on actual behavior through behavioral intention all have 95% confidence intervals that do not contain 0, and their *p*-values are <0.05. This indicates that behavioral intention plays a significant mediating role in the aforementioned paths. Among them, the mediating effect of perceived behavioral control is the strongest (Estimate = 0.152, *p* < 0.001), while perceived barriers demonstrates a significant negative mediation (Estimate = −0.086, *p* < 0.001).

**Figure 3:**
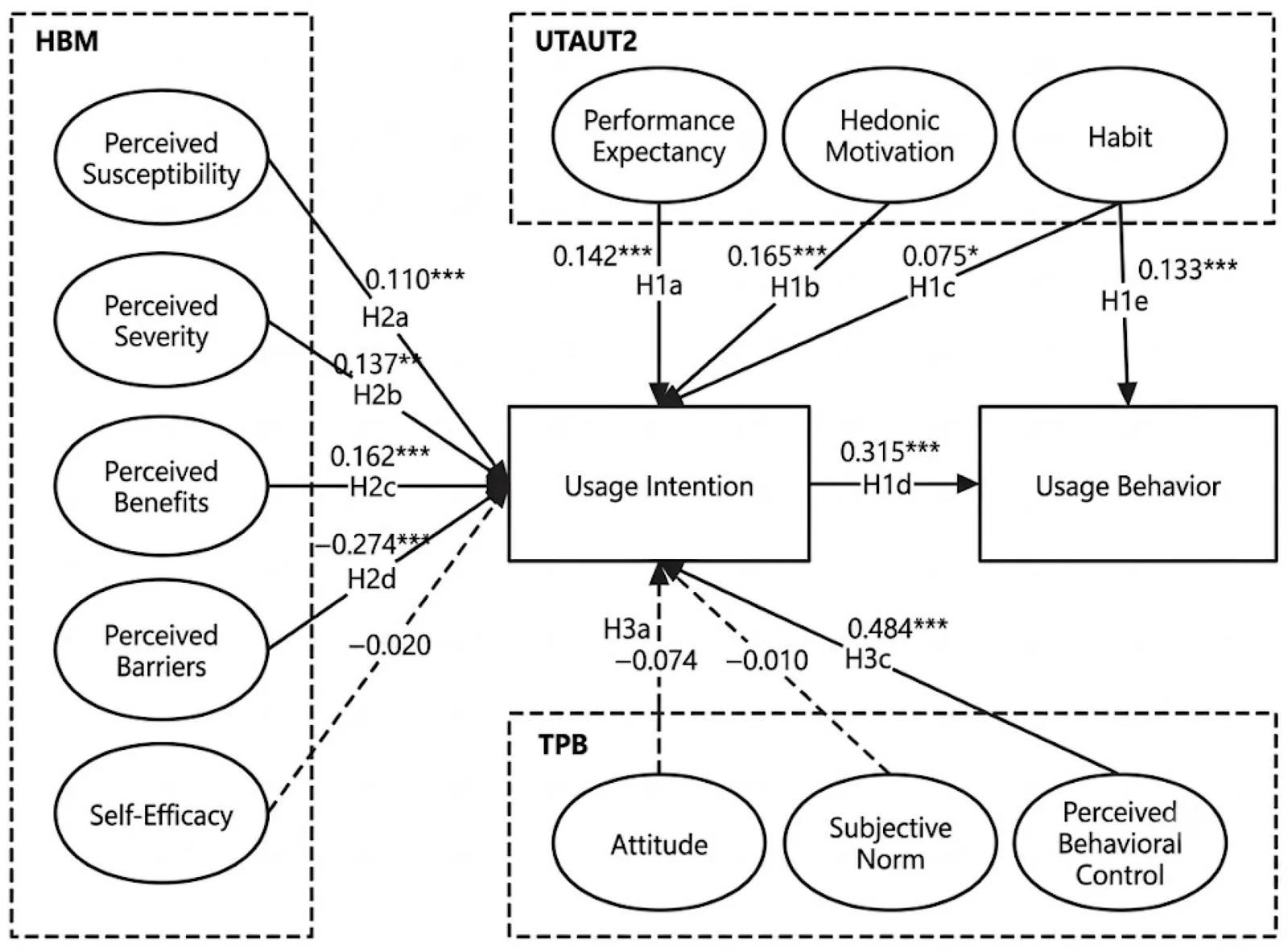
Results of structural model testing. HBM: Health Belief Model; UTAUT2: Unified Theory of Acceptance and Use of Technology 2; TPB: Theory of Planned Behavior; solid lines indicate significant path relationships, while dashed lines represent non-significant paths. * *p* < 0.05, ** *p* < 0.01, *** *p* < 0.001.

Conversely, the indirect effects of habit, self-efficacy, attitude, and subjective norm on actual behavior through behavioral intention have 95% confidence intervals that all contain 0, indicating that the mediating effects in these paths are non-significant. This result aligns with the conclusions of the direct effect tests, further revealing the core pathways of behavioral conversion regarding the use of fitness short videos. Figure 3 shows the results of structural model testing.

## 6. Discussion

The validation of our integrated UTAUT2-HBM-TPB model provides a nuanced deconstruction of the psychological mechanisms governing the transition from sporadic viewing to the sustained usage of fitness short videos. Unlike traditional health interventions that rely on structured environments, the findings here emphasize a shift toward behavioral automation and situational control.

### 6.1. The Primacy of Perceived Behavioral Control: A Paradigm of Instrumental Rationality

The first is that the UTAUT2 model has been applied in recent years to digital fitness. Performance expectancy (β = 0.142, *p* < 0.001) and hedonic motivation (β = 0.165, *p* = 0.001) were the only positive significant predictors; therefore, it can be concluded that the primary reasons for users engaging in fitness knowledge acquisition and video consumption are the basic usefulness of such content and the pleasure derived from it. Although the above reasons can provide a motivational foundation, they are rather indirect indicators of behavioral intent in the current era, and perceived behavioral control (PBC) stands out as the main predictor of people’s intention to use it (β = 0.524, *p* < 0.001) (Ajzen, 1991). Overall, these findings indicate that users’ continued engagement with fitness short videos is primarily driven by their perceived ability to conveniently integrate such behaviors into their daily routines, rather than by health motivations alone.

This finding aligns with the seminal work of Ajzen (1991), which posits that PBC is a primary determinant of behavioral intention; however, our results extend this by demonstrating that in the algorithm-mediated environment of short videos, PBC functions as a “situational facilitator” that lowers immediate psychological barriers, rather than merely reflecting long-term resource assessment as suggested in traditional TPB models. Given the background of fitness short videos, people’s choice to participate in them is less about long-term health goals and more about avoiding inconvenience in daily life. This aligns with recent findings in mobile application adoption, such as the analysis of perceived ease of use and security on mobile banking adoption (Addula, 2024). Such studies demonstrate that digital platform adoption is heavily reliant on structural ease and perceived friction, reinforcing the argument that in mobile environments, perceived behavioral control dominates over traditional, long-term health beliefs. When people feel that they have more control over their own lives due to the low barrier to entry of mobile platforms, they will be more likely to continue using them even without favorable health beliefs. This suggests that in the fragmented digital ecosystem, the “can I do it now” factor has taken the place of an assessment of “should I do it for my future”, and people are no longer motivated by intrinsic motives as much as they were under older theories of health behavior (Ajzen, 1991; Bandura, 1997; Rosenstock, 1974).

From a theoretical perspective, continuous participation is mainly motivated by other reasons besides one’s own attitude or health consciousness in health behavior research (Ajzen, 1991; Conner & Armitage, 1998). Most studies of digital health intervention so far have focused on how to use people’s beliefs about health and self-regulation to keep healthy (Bandura, 1997; Janz & Becker, 1984). Based on the above results, it can be concluded that in the algorithm-mediated environment of short-video consumption, the psychological structure of continuous use has changed; users now regard fitness content as an easy activity that can be done quickly, rather than an objective for maintaining good health. This is in line with the new theory of “situational engagement” in the environment of mobile media (Limayem et al., 2007; Xiao et al., 2026), so to maintain behavior continuity, one needs to reduce environmental friction rather than stimulate one’s own motivation.

### 6.2. The Dual-Path Mechanism of Habit: Cognitive Bypass and Intention Decoupling

In addition, the two-path mechanism of habit (HB) is also a kind of “cognitive bypass” (Limayem et al., 2007). Although the formation of habits is generally based on conscious intention, our results show that habits can also directly motivate people to use them in reality without deliberation. As a result, people have shifted from deliberate thought to automatic response in order to get fit because of the nature of algorithmic media and endless scrolling. This “intention bypass” is very damaging to the continuous development of digital health promotion, and, as a result, people’s efforts in exercise have been reduced and have become automatic daily habits.

From a theoretical perspective, the two-path mechanism identified in this study expands the theoretical role of habit within the UTAUT2 framework. Habit is often regarded as a modifier of intention to engage in behavior in the original UTAUT2 model that can strengthen the effect of intention (Limayem et al., 2007; Vella et al., 2023). However, based on the above, it can be seen that habit is a direct behavioral trigger and requires less willpower. This is in line with the difference that Gardner et al. (2023) made between habitual instigation and habitual execution, but it goes further to show that in algorithmically curated environments, the environmental cue–behavior link is too close, and thus the motivational mediation of intention is effectively bypassed.

For digital health professionals, it may be that nurturing a sense of habit will be more effective than stimulating conscious motivation; thus, it is necessary to adopt the opposite approach of conventional health education.

### 6.3. Asymmetry in Health Beliefs: The Loss Aversion Effect in Digital Fitness

Perhaps the most theoretically significant result is that there is an observed asymmetry in the constructs of the Health Belief Model (HBM), and the inhibitory effect of perceived barriers (PBar) was significantly greater than the promotional effect of perceived benefits (PB). To statistically validate this asymmetry, a pairwise parameter comparison using the Critical Ratios for Differences between Parameters provided by AMOS (Byrne, 2016) was conducted. The test confirmed that the absolute difference between the unstandardized path coefficients of perceived barriers and perceived benefits is highly significant (C.R. = −7.131, *p* < 0.001). Based on the ideas of Prospect Theory, it is proposed that there will be significant “loss aversion” in the field of digital health (Kahneman & Tversky, 1979). People are highly sensitive to “friction”, such as how difficult it is for them to operate a thing or the length of a video. Although it is known that there will be some health benefits, there are still minor problems that can lead to an immediate loss of the wish to use it. It can be seen that the psychological barrier to digital fitness is not a lack of health consciousness, but rather a sensitive negative friction; therefore, it is more urgent to address this obstacle than to promote the advantages and ensure the continuous use of the public.

From a practical perspective, these findings suggest that reducing platform-related friction may be more effective than simply emphasizing health benefits when designing digital fitness interventions.

This asymmetry contradicts the symmetrical treatment of benefits and barriers in conventional HBM applications, where both constructs are assumed to exert comparable but opposite influences on health behavior (Janz & Becker, 1984; Rosenstock, 1974). Our results suggest that in the context of discretionary digital health engagement, the psychological calculus is fundamentally skewed toward risk avoidance. This finding is consistent with Kahneman and Tversky’s (1979) proposition that losses loom larger than gains, but extends it to the domain of digital health by identifying the specific forms of “micro-losses”—temporal cost, cognitive load, physical discomfort—that disproportionately deter sustained usage. Prior research on mobile health apps has similarly noted the primacy of usability barriers over perceived utility (Angosto et al., 2020; Yang & Koenigstorfer, 2021), but our study provides the first systematic evidence that this barrier-dominance effect holds specifically within the short-form video ecosystem, where the expectation of instantaneous gratification further amplifies sensitivity to friction.

### 6.4. The Empirical Failure of Social and Attitudinal Predictors: De-Socialization and De-Skilling in Algorithmic Consumption

Empirically, it has been shown that these three variables (self-efficacy, attitude, and subjective norm) will not predict whether someone intends to do something at a certain time. Rather than viewing these non-significant results as definitive proof, we hypothesize that they may reflect a manifestation of the “de-socialization” and “de-skilling” of algorithmic media consumption. Given that the consumption environment for fitness short videos is often private and individualized, operated by personalized recommendation algorithms instead of social graphs (Zhang et al., 2024), we speculate that the regulating power of peer pressure and social norms in the Theory of Planned Behavior appears to be weakened. The design of short-form content in a micro-module can also break down a complicated fitness plan into many small steps; thus, a person does not need a high level of skill and is not intimidated by the fear of failure. Consistent with previous technology adoption research, users’ engagement with digital platforms is often driven by convenience, perceived ease of use, and situational accessibility rather than solely by health beliefs or emotional attachment (Limayem et al., 2007; Venkatesh et al., 2012). The kind of “technology adoption” known as engagement is driven by urgent circumstances and convenience; it is not an emotional habit or an evaluation of one’s own physical condition.

SN and ATT have no effect, which is not in line with the TPB. In traditional applications of TPB in the health and technology fields, attitude has consistently been one of the main factors predicting behavior (Ajzen, 1991; Conner & Armitage, 1998), and subjective norm usually has a moderately positive effect, especially in collectivistic cultures (Zhang et al., 2017). Based on the above results, we hypothesize that the algorithmic mediation of content discovery may have altered the conventional routes for socialization and psychology. When the distribution of content is based on recommendation algorithms instead of social networks, and the type of engagement is ephemeral, there is a theoretical failure of attitudes and normative bases for rational behavior, and consumption takes the form of low-stake enjoyment rather than deliberate life orientation. Recently, some studies have put forward the idea of a “de-socialization” effect in the environment of personalized media (Zhang et al., 2024). While our observations align with this concept, they should be strictly treated as exploratory hypotheses rather than confirmed empirical facts in the context of digital health behavior.

There is also a problem that people lack self-efficacy theoretically. Bandura proposed in his original theory (Bandura, 1997) that the main way people turn their beliefs about their own capabilities into behavior is by means of self-efficacy. Based on the results we have obtained, it can be seen from the platform design of how to break down complicated behavior into a series of small steps that are easy for people to follow. The user does not need to be able to do an entire workout anymore; they only need to be able to swipe and follow a 15 s demonstration. De-skilling refers to a basic change in the link between the perception of ability and behavior, and when there is severe behavioral reduction, it does not meet the general applicability requirements for the theory of self-efficacy. However, it is crucial to acknowledge that our study did not directly measure specific algorithmic features; thus, these explanations regarding algorithmic de-socialization and de-skilling remain speculative hypotheses.

## 7. Implications

### 7.1. Theoretical Implications

This study makes four main theoretical contributions to the fields of digital health, human–computer interaction (HCI), and behavioral psychology. These contributions address important challenges in integrating technology acceptance theories with health behavior research and provide a more comprehensive framework for understanding continued fitness short video usage.

First, it links the previous division of technology acceptance models, health psychology frameworks and planned behavior theories by integrating UTAUT2 (Unified Theory of Acceptance and Use of Technology 2), HBM (Health Belief Model) and TPB (Theory of Planned Behavior) into one comprehensive explanation system that has not been used in earlier studies of digital health. In the past, some studies have either considered users to be “passive consumers of IT artifacts” and used only UTAUT2 (Venkatesh et al., 2012; Zhang et al., 2017), or as “active participants in health interventions” and focused only on HBM (H. M. Kim et al., 2023; Rosenstock, 1974); these studies have not paid attention to the dual status of users of digital health (J. Kim & Park, 2012). Through the combination of the three models, this study aims to provide more specific and strong support for understanding the reasons why people continue to use digital health applications; that is to say, it is not only about one’s attitude towards the technology (such as performance expectation, effort expectation) or beliefs about their own health (such as perceived susceptibility, perceived benefits), but also about the combination of both. Recent studies have similarly emphasized the critical role of these integrated theoretical perspectives in explaining users’ acceptance of emerging digital technologies such as artificial intelligence-driven platforms (Ma & Huo, 2023). The above attempts to address the serious lack of research mentioned in the recent review (Angosto et al., 2020), and it is hoped that more comprehensive systems encompassing both technology and psychology for the use and management of digital health in active life will be developed.

Secondly, based on the above, it is not possible to apply the traditional behavioral model to the environment of algorithmic media, and thus the classical constructs of subjective norm, attitude and self-efficacy cannot predict the persistence of digital health. Previously, it was thought that the main reasons affecting people’s health behavior were social influence (subjective norms) and one’s own attitudes towards this behavior; however, in the environment of algorithm-mediated digital health, it has been found that the traditional type of social regulation of behavior, such as peer pressure and the need for social approval, has been replaced by algorithmic regulation, including personalized recommendations and content curation.

Therefore, we need to rethink how we define human agency and social influence in the context of digital health; in traditional social environments, social norms are clear and unchanging, but in the world driven by algorithms, they are more subtle and fluid, so the original subjective norm is no longer applicable. Therefore, research on behavioral models in the digital environment has also been expanding; it has been found that the old framework needs to be adjusted according to the specific features of algorithm-driven media, and due to the “micro-module” design of short videos, the execution barrier has fallen significantly, and internal self-efficacy is no longer the bottleneck.

Thirdly, in accordance with the framework of behavioral economics and through mathematical demonstrations of HBM theory, this paper will examine “loss aversion” (Kahneman & Tversky, 1979) to address the problem of a high dropout rate for e-health platforms. Previous research on HBM has focused more on “gain-framed” factors (e.g., perceived benefits of healthy behavior) and has not yet studied the role of loss aversion; in line with the main idea of behavioral economics, people are more motivated by losses than by gains. Integration of behavioral economics and Health Belief Models has expanded the system of theoretical construction for research on digital health and offered new ways to understand the reasons why people continue to maintain good health in the digital age. Integration of behavioral economics and Health Belief Models has expanded the system of theoretical construction for research on digital health and offered new ways to understand the reasons why people continue to maintain good health in the digital age.

Finally, this study contributes to technology acceptance research by validating the dual-path mechanism of habit. In the past, many studies have often treated habits as moderators or precursors to intention, but based on our results, it may be that habits directly lead to behavior and are not mediated by deliberate intentions. Cognitive bypass is the phenomenon that, with the passage of time, people’s digital health behaviors have become increasingly reflexive behaviors triggered by environmental cues and have lost their sense of free will.

### 7.2. Practical Implications

Based on the findings of this study, several practical implications can be drawn for digital fitness platform developers, public health practitioners, and policymakers. The results indicate that perceived behavioral control plays the most important role in predicting behavioral intention, while perceived barriers remain a key obstacle to continued fitness short video usage.

The first priority for digital fitness platform developers is to reduce users’ perceived barriers and improve the ease of accessing fitness-related content. Consistent with the structural equation modeling results, perceived barriers showed a significant negative effect on behavioral intention (β = −0.343, *p* < 0.001). Therefore, reducing users’ perceived barriers may be an effective strategy for improving user control, continued engagement, and long-term participation in fitness short-video platforms.

Based on the research findings, digital fitness platforms may place less emphasis on traditional social-sharing strategies and instead focus on facilitating habitual use based on users’ behavioral patterns. Since our data indicates that ‘lack of time’ is a primary perceived barrier (PBar), platforms should allow users to seamlessly integrate short workouts into their fragmented schedules without requiring complex setup, rather than relying on unmeasured external triggers.

Finally, aligning with the non-significant effects of social influence observed in our data, the public health department and the platform should provide personalized and accessible services. For example, rather than proposing unmeasured technologies like AR, platforms should directly address the equipment and movement barriers identified in our survey. Offering adjustable playback speeds and clear interfaces for small screens can provide practical support to guide us on how to exercise properly, allowing users to focus on personal health goals without the pressure of social interaction.

Based on the findings of this study, traditional social influence (subjective norm) showed limited effects on behavioral intention. Therefore, digital fitness platforms may place greater emphasis on reducing perceived barriers and enhancing users’ perceived behavioral control rather than relying primarily on social interaction features. Such strategies may help facilitate continued engagement with fitness short videos.

## 8. Conclusions, Limitations, and Future Research

In conclusion, this study developed and validated an integrated framework combining UTAUT2, the Health Belief Model (HBM), and the Theory of Planned Behavior (TPB) to explain users’ behavioral intention and continued usage of fitness short videos. The findings indicate that perceived behavioral control, perceived barriers, performance expectancy, hedonic motivation, and habit are important determinants of behavioral intention and usage behavior, whereas attitude, subjective norm, and self-efficacy showed no significant effects. These findings contribute to the understanding of technology-supported health behaviors and provide both theoretical insights and practical implications for promoting sustained engagement with digital fitness platforms.

The above results have been obtained, but this study also has the following shortcomings: First, because this study employed a cross-sectional survey design, it was not possible to observe changes in user habits over time or establish causal relationships. Furthermore, convenience and snowball sampling rather than probability sampling were adopted. Although this approach facilitated efficient data collection from fitness short video users, it may have introduced selection bias and limited the representativeness of the sample. Therefore, the generalizability of the findings should be interpreted with caution. In addition, the theoretical model did not include algorithm-related variables, such as perceived personalization, user interface details, and information overload, which may influence users’ behavioral intentions. Because we did not empirically measure specific algorithmic features, our discussion regarding algorithmic “de-socialization” and “de-skilling” remains exploratory and should be treated as a hypothesis rather than a confirmed conclusion. Finally, the sample of this study is not strictly representative of the entire population, as the respondents primarily consist of young and middle-aged individuals. Moreover, the empirical data was collected exclusively within a Chinese context. Given the specificities of cultural backgrounds and localized digital fitness ecosystems, the results do not claim to be automatically transferable to other cultural settings outside of China.

To address these limitations, future research should aim to validate this integrated model across more diverse age groups and broader cross-cultural contexts. Furthermore, future studies should adopt longitudinal designs or ecological momentary assessments to capture dynamic causal relationships, and expand the theoretical model by incorporating algorithm-specific variables, thereby developing a more universal framework for digital health promotion.

## Figures and Tables

**Table 1:** Measurements for constructs.

Construct	Measurement Items	Sources
Performance Expectancy (PE)	PE1: Watching fitness short videos allows me to acquire fitness knowledge more efficiently. PE2: Fitness short videos can meet my daily needs for fitness and exercise. PE3: Fitness short videos can help me achieve my personal fitness goals. PE4: Overall, fitness short videos are indeed very helpful for me to achieve my fitness/health goals.	(Venkatesh et al., 2012)
Hedonic Motivation (HM)	HM1: Watching fitness short videos makes me feel pleasant and relaxed. HM2: Watching fitness short videos is an enjoyment in itself, rather than just for exercise. HM3: Watching fitness short videos makes me feel excited and energetic. HM4: I think watching fitness short videos is an interesting way of entertainment.	(Venkatesh et al., 2012)
Habit (HB)	HB1: I have a habit of exercising by following fitness short videos. HB2: Fitness short videos have become an important part of my life. HB3: Persisting in checking in with fitness short videos has become my fixed habit. HB4: I would feel uncomfortable if I don’t use fitness short videos for a period of time.	(Limayem et al., 2007)
Perceived Susceptibility (PS)	PS1: I think if I don’t persist in exercising, health problems (such as physical decline, weight gain) will easily occur. PS2: I feel that my health is easily affected by a lack of exercise (such as easy fatigue, declined immunity). PS3: I worry that my lifestyle may increase the risk of chronic diseases.	(Champion, 1984; Rosenstock, 1974)
Perceived Severity (PSev)	PSev1: I believe that weight gain will bring serious health consequences. PSev2: Obesity-related diseases will have a significant impact on my quality of life. PSev3: If no action is taken, I may face serious health problems.	(Champion, 1984; Rosenstock, 1974)
Perceived Benefits (PB)	PB1: Watching fitness short videos can help me manage my weight well. PB2: The exercise methods provided by fitness short videos are helpful for improving physical health. PB3: Acquiring fitness knowledge through fitness short videos is more convenient and effective than other methods.	(Champion, 1984; Rosenstock, 1974)
Perceived Barriers (PBar)	PBar1: I often find it difficult to consistently watch fitness short videos due to a lack of time. PBar2: I feel that the movements in fitness short videos are sometimes too difficult to follow. PBar3: Technical limitations (e.g., searching issues) or equipment issues (e.g., small screen) reduce my willingness to use fitness short videos.	(Champion, 1984; Rosenstock, 1974)
Self-Efficacy (SE)	SE1: I am confident that I can exercise following the fitness short videos. SE2: I believe I can persist in exercising with fitness short videos. SE3: Even when facing difficulties, I can maintain the habit of exercising with fitness short videos.	(Bandura, 1997; Champion, 1999)
Attitude (ATT)	ATT1: I think using fitness short videos for exercise is a positive behavior. ATT2: I feel that fitness short videos are beneficial to me. ATT3: I like to use fitness short videos for fitness learning or exercise. ATT4: Overall, I hold a positive attitude towards using fitness short videos.	(Ajzen, 1991; Taylor & Todd, 1995)
Subjective Norm (SN)	SN1: People who are important to me think I should use fitness short videos. SN2: My family or friends would encourage me to use fitness short videos. SN3: Seeing many people using fitness short videos on social media makes me willing to use them too.	(Ajzen, 1991; Taylor & Todd, 1995)
Perceived Behavioral Control (PBC)	PBC1: I have the ability to exercise following the movements in fitness short videos. PBC2: I have enough time and resources to use fitness short videos. PBC3: I think I can easily integrate fitness short videos into my daily life.	(Ajzen, 1991; Taylor & Todd, 1995)
Behavioral Intention (BI)	BI1: I intend to continue using fitness short videos in the future. BI2: I am willing to recommend fitness short videos to people around me in the future. BI3: I will prioritize short videos as my preferred way to obtain fitness guidance. BI4: I want to express positive evaluations of fitness short videos to others.	(Ajzen, 1991; Venkatesh et al., 2003; Venkatesh et al., 2012)
Usage Behavior (UB)	UB1: Watching/exercising with fitness short videos has become a fixed weekly schedule for me. UB2: The frequency and duration of my exercise using fitness short videos each week are relatively stable. UB3: I will adjust my exercise plan and movement details based on the content in fitness short videos.	(Ajzen, 1991; Venkatesh et al., 2003; Venkatesh et al., 2012)

**Table 2:** Demographic characteristics and usage patterns of the respondents (*N* = 550).

Measure	Category	Frequency	Percentage (%)
Gender	Male	240	43.6
	Female	310	56.4
Age	≤18	32	5.8
	19–25	228	41.5
	26–35	203	36.9
	36–45	69	12.5
	≥46	18	3.3
Education	High school or below	46	8.4
	Junior college	156	28.4
	Undergraduate	285	51.8
	Master’s degree or above	63	11.5
Usage Frequency	0–1 time/week	95	17.3
	2–4 times/week	296	53.8
	5–6 times/week	90	16.4
	≥7 times/week	69	12.5
Session Length	<10 min	109	19.8
	10–30 min	292	53.1
	30–60 min	100	18.2
	>60 min	49	8.9

Note: Data are presented as frequencies and percentages (%). *N* = 550.

**Table 3:** Reliability and validity assessment of the measurement model.

Construct	Cronbach’s α	CR	AVE
Performance Expectancy (PE)	0.887	0.851	0.656
Hedonic Motivation (HM)	0.860	0.866	0.62
Habit (HB)	0.888	0.889	0.667
Perceived Susceptibility (PS)	0.835	0.84	0.635
Perceived Severity (PSev)	0.804	0.815	0.596
Perceived Benefits (PB)	0.869	0.87	0.691
Perceived Barriers (PBar)	0.829	0.829	0.618
Self-Efficacy (SE)	0.864	0.865	0.682
Attitude (ATT)	0.846	0.848	0.583
Subjective Norm (SN)	0.853	0.854	0.662
Perceived Behavioral Control (PBC)	0.794	0.796	0.566
Behavioral Intention (BI)	0.846	0.814	0.523
Usage Behavior (UB)	0.859	0.855	0.663

Note: Standardized factor loadings are reported. CR = composite reliability; AVE = average variance extracted.

**Table 4:** Pearson correlation matrix of the latent variables.

	PE	HM	HB	PS	PSev	PB	PBar	SE	ATT	SN	PBC	BI	UB
PE	1												
HM	0.320 **	1											
HB	0.231 **	0.354 **	1										
PS	0.129 **	0.296 **	0.630 **	1									
PSev	0.243 **	0.276 **	0.305 **	0.327 **	1								
PB	0.485 **	0.243 **	0.188 **	0.188 **	0.360 **	1							
PBar	0.034	0.313 **	0.121 **	0.202 **	0.128 **	0.025	1						
SE	0.253 **	0.265 **	0.168 **	0.238 **	0.274 **	0.419 **	0.098 *	1					
ATT	0.206 **	0.330 **	0.240 **	0.281 **	0.520 **	0.208 **	0.084 *	0.226 **	1				
SN	0.276 **	0.272 **	0.205 **	0.209 **	0.243 **	0.328 **	0.047	0.501 **	0.335 **	1			
PBC	0.328 **	0.290 **	0.421 **	0.392 **	0.347 **	0.299 **	0.169 **	0.238 **	0.309 **	0.329 **	1		
BI	0.389 **	0.307 **	0.400 **	0.355 **	0.365 **	0.380 **	−0.096 *	0.239 **	0.264 **	0.270 **	0.547 **	1	
UB	0.282 **	0.250 **	0.270 **	0.214 **	0.291 **	0.393 **	−0.01	0.298 **	0.249 **	0.338 **	0.211 **	0.328 **	1

Note: Values represent Pearson correlation coefficients between latent variables. ** *p* < 0.01; * *p* < 0.05.

**Table 5:** Model goodness-of-fit test results *(N* = 550).

Fit Indices	Recommended Criteria	Model Results	Conclusion	Source
χ^2^/df	<3 (Ideal); <5 (Acceptable)	3.538	Good Fit	(Hu & Bentler, 1999)
GFI	>0.90 (Ideal); >0.80 (Basic)	0.773	Marginal Fit	(Hair et al., 2010)
AGFI	>0.90 (Ideal); >0.80 (Basic)	0.743	Marginal Fit	(Hair et al., 2010)
RMR	<0.05 (Ideal); <0.08 (Good)	0.19	High *	(Hu & Bentler, 1999)
CFI	>0.90 (Ideal); >0.80 (Basic)	0.834	Basic Fit	(Hu & Bentler, 1999)
TLI	>0.90 (Ideal); >0.80 (Basic)	0.82	Basic Fit	(Hu & Bentler, 1999)
IFI	>0.90 (Ideal); >0.80 (Basic)	0.835	Basic Fit	(Hair et al., 2010)
NFI	>0.90 (Ideal); >0.80 (Basic)	0.783	Marginal Fit	(Hair et al., 2010)
PNFI	>0.50	0.724	Good Fit	(Mulaik et al., 1989)
PCFI	>0.50	0.77	Good Fit	(Mulaik et al., 1989)

Note: χ^2^/df = chi-square-to-degrees-of-freedom ratio; CFI = comparative fit index; TLI = Tucker–Lewis index; IFI = incremental fit index; PNFI = parsimonious normed fit index; PCFI =parsimonious comparative fit index; GFI = goodness-of-fit index; AGFI = adjusted goodness-of-fit index; NFI = normed fit index; RMR = root mean square residual. * Indicates a high residual value that notably exceeds the recommended criteria of <0.08.

**Table 6:** Structural equation modeling path verification results (*N* = 550).

Structural Path	Estimate (β)	S.E.	C.R.	*p*-Value	Significance
Behavioral Intention ← Perceived Susceptibility	0.11	0.034	3.214	0.001	Significant
Behavioral Intention ← Perceived Severity	0.137	0.05	2.728	0.006	Significant
Behavioral Intention ← Perceived Benefits	0.162	0.05	3.218	0.001	Significant
Behavioral Intention ← Perceived Barriers	−0.343	0.037	−7.331	<0.001	Significant
Behavioral Intention ← Perceived Behavioral Control	0.524	0.054	9.036	<0.001	Significant
Behavioral Intention ← Performance Expectancy	0.142	0.043	3.31	<0.001	Significant
Behavioral Intention ← Hedonic Motivation	0.165	0.051	3.265	0.001	Significant
Behavioral Intention ←Habit	0.075	0.035	2.125	0.034	Significant
Behavioral Intention ← Self-Efficacy	−0.02	0.045	−0.449	0.653	Non-significant
Behavioral Intention ← Attitude	−0.074	0.054	−1.38	0.168	Non-significant
Behavioral Intention ← Subjective Norm	−0.01	0.048	−0.214	0.83	Non-significant
Actual Behavior ← Behavioral Intention	0.315	0.053	5.978	<0.001	Significant
Actual Behavior ← Habit	0.133	0.039	3.428	<0.001	Significant

Note: S.E. = standard error; C.R. = critical ratio. The coefficients reported in the “Estimate (β)” column are standardized path coefficients (β), representing the expected change in the dependent variable (in standard deviation units) for a one-standard-deviation change in the independent variable.The arrow (←) indicates the direction of the hypothesized path, pointing from the independent variable to the dependent variable.

**Table 7:** Mediation effect test results (bootstrap = 5000).

Mediation Path	Indirect Estimate	Lower 95% CI	Upper 95% CI	*p*-Value	Result
Perceived Susceptibility → BI → UB	0.035	0.005	0.072	0.019	Significant
Perceived Severity → BI → UB	0.043	0.003	0.099	0.035	Significant
Perceived Benefits → BI → UB	0.051	0.01	0.113	0.014	Significant
Perceived Barriers → BI → UB	−0.086	−0.127	−0.053	<0.001	Significant
Self-Efficacy → BI → UB	−0.006	−0.044	0.03	0.707	Non-significant
Performance Expectancy → BI → UB	0.045	0.008	0.093	0.017	Significant
Hedonic Motivation → BI → UB	0.052	0.012	0.103	0.009	Significant
Habit → BI → UB	0.024	−0.008	0.063	0.141	Non-significant
Attitude → BI → UB	−0.023	−0.073	0.021	0.287	Non-significant
Subjective Norm → BI → UB	−0.003	−0.044	0.038	0.828	Non-significant
Perceived Behavioral Control → BI → UB	0.152	0.099	0.227	<0.001	Significant

Note: Bootstrap sample size = 5000. Indirect estimates represent standardized path coefficients (β) for the indirect effects. CI = confidence interval.The arrow (→) indicates the direction of the hypothesized path.

## Data Availability

The datasets used in the current study are available from the corresponding author upon reasonable request.

## Appendix A. Measurement Items and Standardized Factor Loading

**Construct**	**Measurement Items**	**Standardized Loading**
Performance Expectancy (PE)	PE1: Watching fitness short videos allows me to acquire fitness knowledge more efficiently.	0.775
	PE2: Fitness short videos can meet my daily needs for fitness and exercise.	0.827
	PE3: Fitness short videos can help me achieve my personal fitness goals.	0.827
	PE4: Overall, fitness short videos are indeed very helpful for me to achieve my fitness/health goals.	0.827
Hedonic Motivation (HM)	HM1: Watching fitness short videos makes me feel pleasant and relaxed.	0.672
	HM2: Watching fitness short videos is an enjoyment in itself, rather than just for exercise.	0.846
	HM3: Watching fitness short videos makes me feel excited and energetic.	0.808
	HM4: I think watching fitness short videos is an interesting way of entertainment.	0.812
Habit (HB)	HB1: I have a habit of exercising by following fitness short videos.	0.805
	HB2: Fitness short videos have become an important part of my life.	0.827
	HB3: Persisting in checking in with fitness short videos has become my fixed habit.	0.81
	HB4: I would feel uncomfortable if I don’t use fitness short videos for a period of time.	0.824
Perceived Susceptibility (PS)	PS1: I think if I don’t persist in exercising, health problems (such as physical decline, weight gain) will easily occur.	0.795
	PS2: I feel that my health is easily affected by a lack of exercise (such as easy fatigue, declined immunity).	0.72
	PS3: I worry that my lifestyle may increase the risk of chronic diseases.	0.869
Perceived Severity (PSev)	PSev1: I believe that weight gain will bring serious health consequences.	0.821
	PSev2: Obesity-related diseases will have a significant impact on my quality of life.	0.788
	PSev3: If no action is taken, I may face serious health problems.	0.703
Perceived Benefits (PB)	PB1: Watching fitness short videos can help me manage my weight well.	0.848
	PB2: The exercise methods provided by fitness short videos are helpful for improving physical health.	0.823
	PB3: Acquiring fitness knowledge through fitness short videos is more convenient and effective than other methods.	0.823
Perceived Barriers (PBar)	PBar1: I often find it difficult to consistently watch fitness short videos due to a lack of time.	0.777
	PBar2: I feel that the movements in fitness short videos are sometimes too difficult to follow.	0.767
	PBar3: Technical limitations (e.g., searching issues) or equipment issues (e.g., small screen) reduce my willingness to use fitness short videos.	0.814
Self-Efficacy (SE)	SE1: I am confident that I can exercise following the fitness short videos.	0.834
	SE2: I believe I can persist in exercising with fitness short videos.	0.8
	SE3: Even when facing difficulties, I can maintain the habit of exercising with fitness short videos.	0.843
Attitude (ATT)	ATT1: I think using fitness short videos for exercise is a positive behavior.	0.774
	ATT2: I feel that fitness short videos are beneficial to me.	0.743
	ATT3: I like to use fitness short videos for fitness learning or exercise.	0.764
	ATT4: Overall, I hold a positive attitude towards using fitness short videos.	0.772
Subjective Norm (SN)	SN1: People who are important to me think I should use fitness short videos.	0.858
	SN2: My family or friends would encourage me to use fitness short videos.	0.752
	SN3: Seeing many people using fitness short videos on social media makes me willing to use them too.	0.828
Perceived Behavioral Control (PBC)	PBC1: I have the ability to exercise following the movements in fitness short videos.	0.762
	PBC2: I have enough time and resources to use fitness short videos.	0.738
	PBC3: I think I can easily integrate fitness short videos into my daily life.	0.757
Behavioral Intention (BI)	BI1: I intend to continue using fitness short videos in the future.	0.758
	BI2: I am willing to recommend fitness short videos to people around me in the future.	0.707
	BI3: I will prioritize short videos as my preferred way to obtain fitness guidance.	0.727
	BI4: I want to express positive evaluations of fitness short videos to others.	0.698
Usage Behavior (UB)	UB1: Watching/exercising with fitness short videos has become a fixed weekly schedule for me.	0.843
	UB2: The frequency and duration of my exercise using fitness short videos each week are relatively stable.	0.748
	UB3: I will adjust my exercise plan and movement details based on the content in fitness short videos.	0.848
Note: Standardized factor loadings are reported for each measurement item.		
